# La nécrose de la hanche : Une complication grave de l'injection intrafessière de sels de quinine à Bangui, République centrafricaine

**DOI:** 10.48327/mtsibulletin.2021.120

**Published:** 2021-08-13

**Authors:** M. Onimus, D.S Ouaimon

**Affiliations:** 1 Faculté de médecine de Besançon, Université de Franche Comté, 25000 Besançon, France. Centre de rééducation pour handicapés moteurs, BP 2522, Bangui, République centrafricaine.; 2 Service de chirurgie infantile, Complexe pédiatrique, Centre hospitalo-universitaire de Bangui, République centrafricaine.

**Keywords:** Séquelles d'injection intrafessière, Coxopathie, Enfant, Nécrose cotyle, Toxicité, Quinine, Boiterie, Bangui, République centrafricaine, Afrique subsaharienne, Sequelae of intragluteal injections, Coxopathy, Child, Acetabulum necrosis, Quinine toxicity, Limping, Bangui, Central African Republic, Sub-Saharan Africa

## Abstract

**Introduction:**

L'injection intrafessière de sels de quinine a longtemps été la thérapeutique habituelle de l'accès palustre en zone endémique africaine et elle est encore trop souvent pratiquée. Cependant elle est souvent mal exécutée par du personnel peu ou pas qualifié. Faite trop profondément chez un enfant peu musclé et dénutri, l'injection peut être effectuée dans l'articulation de la hanche.

**Matériel et Méthode:**

À partir d'une série de six observations faites à Bangui, République centrafricaine, les auteurs analysent les aspects cliniques et radiographiques de cette complication qui semble non décrite dans la littérature.

**Résultats:**

La toxicité de la quinine est responsable d'une nécrose atteignant la tête fémorale et le cotyle, entrainant une gêne fonctionnelle notable avec une articulation douloureuse, enraidie en adduction, avec un raccourcissement apparent important. La radiographie montre une nécrose épiphysaire subtotale associée à une nécrose du toit du cotyle, avec une ascension du moignon épiphyso-métaphysaire qui garde un aspect grossièrement sphérique et reste encastré dans un néocotyle relativement profond.

**Discussion:**

En milieu africain, ce tableau peut faire évoquer une nécrose fémorale sur drépanocytose ou, plus encore une séquelle d'ostéoarthrite septique dont le tableau radiographique est cependant différent. Les possibilités thérapeutiques se limitent souvent à la prescription d'une mise en décharge partielle.

## Abstract (en)

**Introduction:**

Intramuscular injection of quinine has been for long the most common treatment for malaria in children in endemic areas of Africa, especially sub-Saharan Africa, and remains too often used. However, it is frequently wrongly performed by unqualified people. When administered in a poorly developed or malnourished child, the injection can be done too deeply in the hip joint instead of the gluteus muscle area.

**Materials and Methods:**

The files of 3012 children examined in out-patient clinics in Bangui, Central African Republic, between 2011 and 2020 were reviewed. Sequelae of intramuscular injections were observed in 307 cases, including intraquadricipital injection in 170 cases (56%) and intragluteal injection in 137 cases (44%). The latter included 115 sciatic paralysis and 22 hip sequelae with stiffness, shortening of the limb, limping and pain at walking. In these 22 cases, an intragluteal injection was incriminated by the families. However, 16 files were considered as insufficient because of imprecise history or because poor quality or no radiograph was available. Although suspected of being hip necrosis, these files were excluded. Six cases presented specific clinical pictures and interpretable radiographs and were included in this study.

**Results:**

The clinical and the radiographic aspects of this severe complication apparently not documented in the literature are analyzed. The well-known toxicity of quinine may be responsible of a necrosis involving both the femoral head and the acetabular roof, resulting in a painful joint, stiff in adduction, with limping and with an apparent marked shortening of the lower limb. Radiographs show a subtotal femoral head necrosis associated to an acetabular roof necrosis with an upward displacement of the epiphyseo-metaphysal femoral stump, the latter keeping a roughly spherical aspect and remaining well-covered and fitted in a relatively deep neo-acetabulum.

**Discussion:**

In the African background, this picture of coxopathy occurring in childhood may suggest an avascular necrosis of the femoral head complicating a sickle-cells disease, or above all sequelae of septic osteoarthritis. Treatments are limited to the prescription of a partial weight bearing of the hip.

**Conclusion:**

Although no irrefutable arguments are existing, the observed clinical and radiographic pictures are sufficiently clear and typical to individualize this severe iatrogenic complication which should be avoided by a good technic or by using the intravenous way when necessary.

## Introduction

En cas de crise de paludisme chez l'enfant en milieu africain, l'injection intramusculaire de sels de quinine, bien qu'actuellement proscrite, reste trop souvent le moyen thérapeutique le plus utilisé dans les dispensaires de quartier, car étant le procédé le moins coûteux et le plus facilement disponible. Quand elle est mal exécutée, l'injection intrafessière peut entraîner une paralysie du nerf sciatique, le plus souvent partielle, intéressant le contingent du nerf fibulaire commun, entraînant une déformation du pied en varus équin. Lorsque l'injection est faite trop profondément, elle peut être effectuée non pas dans la fesse, mais dans l'articulation de la hanche, entraînant une nécrose de l'articulation. Cette complication, qui ne semble pas décrite dans la littérature, est gravissime à cause de son retentissement fonctionnel et à cause de l'absence de possibilités thérapeutiques chez l'enfant.

## Matériel d’étude

L’étude repose sur l'analyse rétrospective de 3 012 dossiers d'enfants vus en consultation d'orthopédie infantile entre les années 2011 et 2020, soit dans le cadre du Centre de Rééducation pour Handicapés Moteurs de Bangui, soit dans le cadre du service de chirurgie infantile du CNHU de Bangui. Des séquelles d'injections intramusculaires ont été retrouvées dans 307 cas. Parmi ceux-ci, il s'agissait de séquelles d'injection intraquadricipitale dans 170 cas, soit 56 %, et de séquelles d'injection intrafessière dans 137 cas, soit 44 %. Ces dernières se répartissaient en 115 cas de paralysies sciatiques et 22 cas de séquelles articulaires au niveau de la hanche avec raideur, raccourcissement du membre inférieur, boiterie et douleur à la marche. Dans ces 22 cas, une injection intrafessière était incriminée par les familles comme étant à l'origine des troubles. Seize d'entre eux présentaient une histoire trop peu précise, pas de clichés radiographiques ou des clichés de qualité trop médiocre, et, bien que suspects de nécrose de la hanche, ils ont été exclus de la série. Six cas présentaient des aspects cliniques suffisamment spécifiques et des radiographies interprétables. Ces cas ont été regroupés et sont l'objet de cette étude.

## Résultats

Six dossiers ont été retenus, représentant 4,4 % des complications observées après injection intrafessière. L’âge moyen lors de la consultation de ces deux garçons et quatre filles était de 16 ans (extrêmes 13 ans – 18 ans) (Tableau I).

**Tableau I:** Analyse des séquelles des 6 cas de nécrose de hanche consécutifs à une injection de sels de quinine Presentation of the 6 cases of hip necrosis observed after intragluteal quinine salt injection

	Âge	Âge en années à l'injection	Amplitude de flexion de hanche	Raccourcissement apparent
1. E.P.	13	6	30°-40°	10 cm
2. P.F.	15	7	20°-30°	10 cm
3. N.J.	18	12	30°-50°	10 cm
4. L.I.	17	12	20°-40°	7 cm
5. T.N.	16	8	10°-30°	10 cm
6. G.E.	17	9	20°-40°	8 cm

La notion d'une injection dans les semaines précédant l'apparition des troubles a été retrouvée dans tous les cas. Le handicap fonctionnel était important: la hanche était enraidie en flexion, en adduction et en rotation nulle; l'adduction entrainait une obliquité pelvienne qui aggravait le raccourcissement, obligeant à un équinisme à l'appui (Fig. 1a). Il existait une discrète mobilité en flexion, mais avec une amplitude limitée à quelques degrés par la douleur.

Figure 1a et 1b.EP (cas n°1). Séquelles probables d'injection intra-articulaire de Quinimax® chez un enfant âgé de 12 ans, encore en phase évolutive.EP (case n° 1). Likely sequelae after intraarticular injection of Quinimax®, still progressing, in a 12 years old patient.

**1a:**
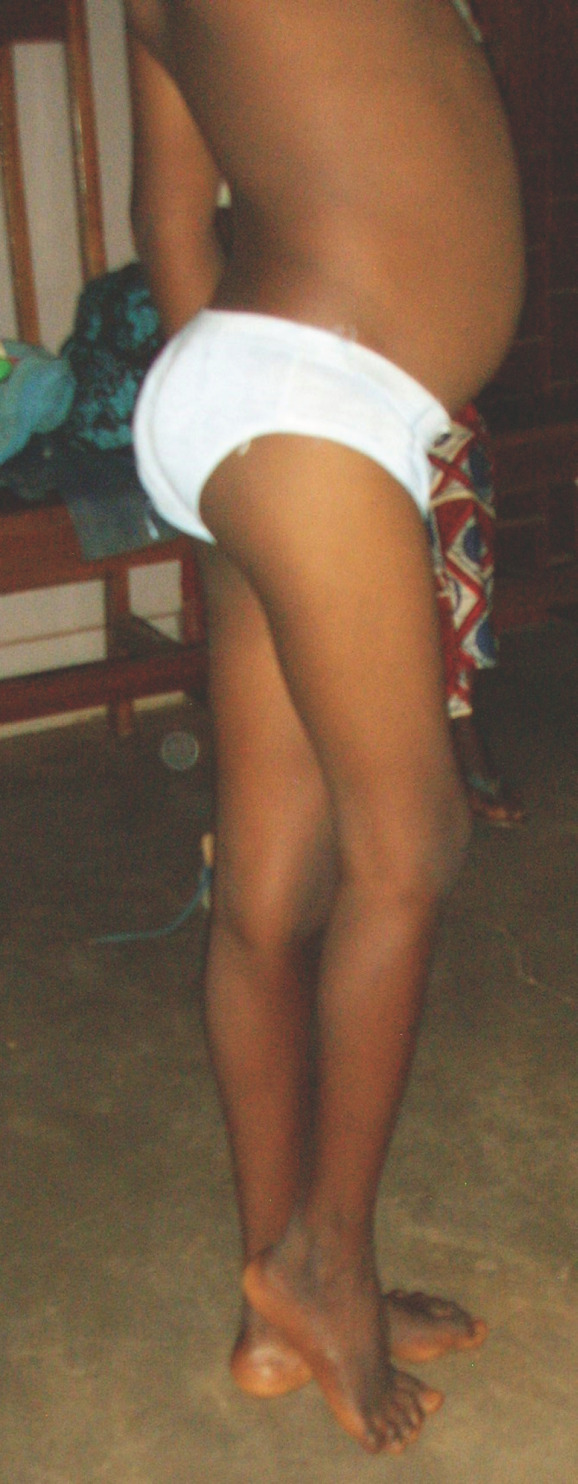
Aspect clinique. La hanche droite est enraidie en flexion; le raccourcissement apparent dépasse 10 cm. Clinical aspect. The right hip is stiff in flexion; apparent shortening is more than 10 cm.

**1b:**
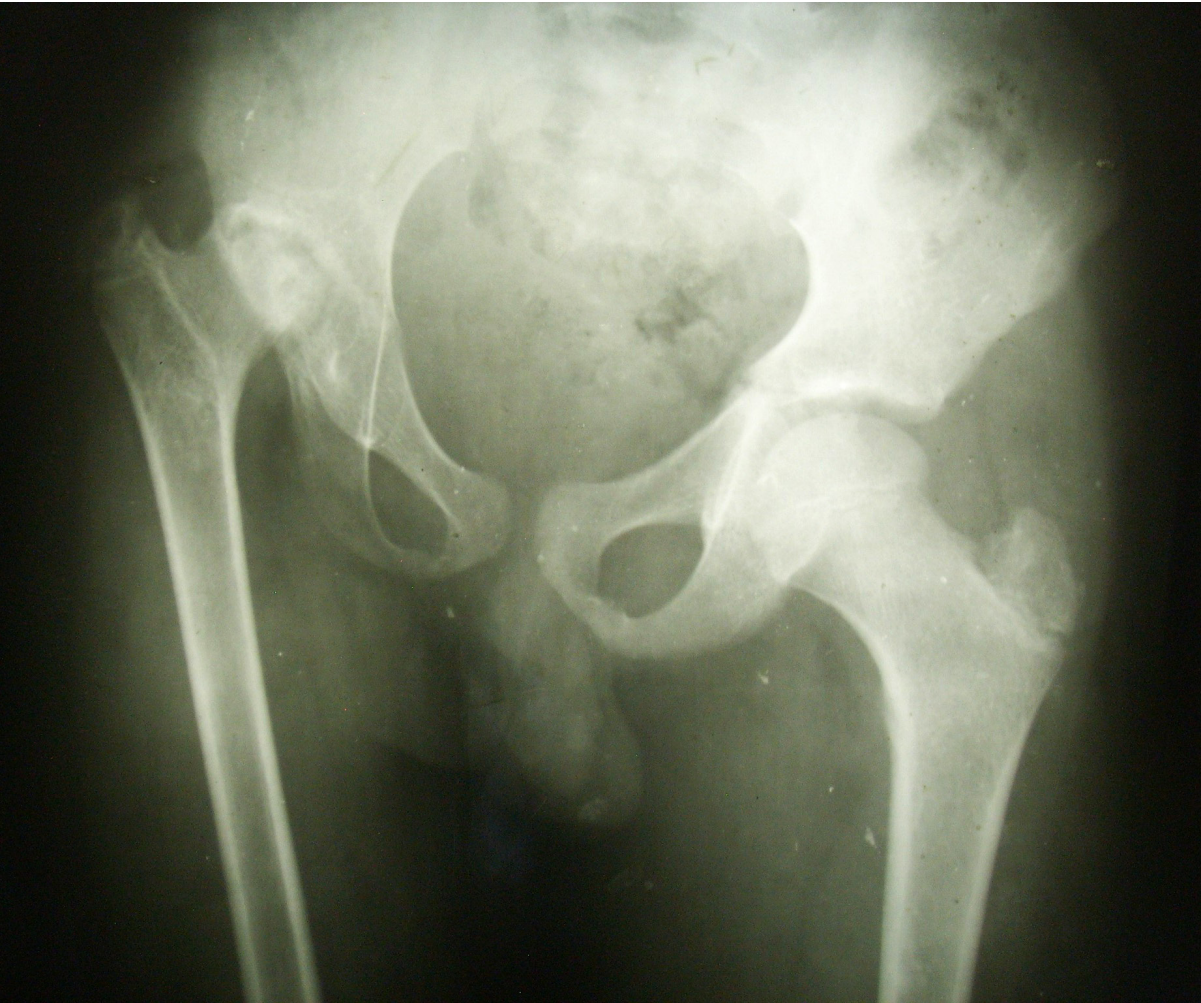
Radiographie. Le tiers supérieur de la tête fémorale est détruit, mais l’épiphyse reste relativement sphérique. Le cotyle est évasé; la tête fémorale est ascensionnée; le cintre cervico-obturateur est nettement perturbé mais le cintre cervico-iliaque est conservé, témoignant de l'absence d'excentration du fémur. Radiograph. The upper third of the femoral head is destroyed, but the epiphysis is still more or less spherical. The acetabulum is widened; the femoral head is displaced upward; there is no subluxation of the femoral head.

**Figure 2:**
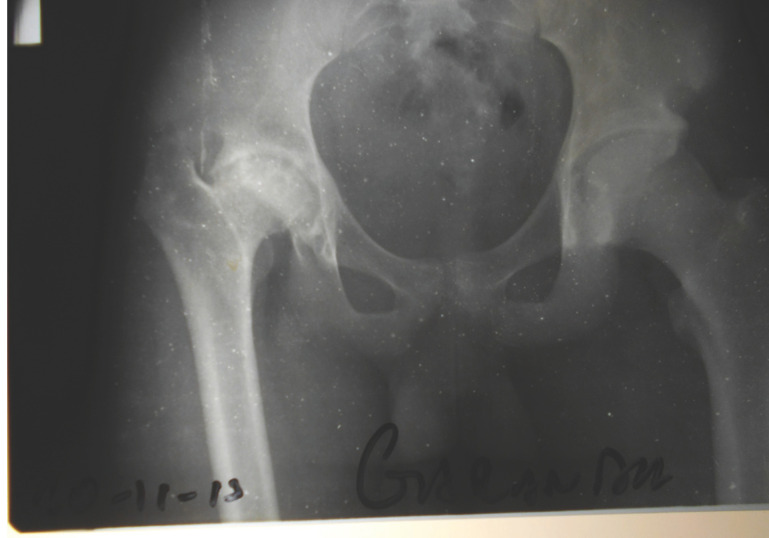
GE (cas n° 6). Nécrose de la hanche droite après injection intrafessière chez un adolescent vu à l’âge de 17 ans, au stade séquellaire. Noter la relative conservation de la sphéricité de la tête fémorale, l’érosion du toit du cotyle, l'absence d'excentration et la profondeur du cotyle. GE (case n°6). Necrosis of the right hip after intragluteal injection in an adolescent seen at 17 at sequelae stage. Notice the sphericity of the femoral head, the osteolysis or the acetabular roof, the absence of hip subluxation and the deepness of the acetabulum.

Les radiographies montraient une nécrose épiphysaire intéressant la quasi-totalité de la tête fémorale, avec dans tous les cas la conservation d'un aspect relativement sphérique du moignon épiphyso-métaphysaire résiduel (Fig. 1b). L'interligne articulaire était présent, mais irrégulièrement pincé. Il existait dans tous les cas une ostéolyse du toit du cotyle avec ascension de la tête fémorale, mais avec conservation de la sclérose osseuse souschondrale (Fig. 2). L'ossification du bord externe du cotyle donnait l'aspect d'un cotyle relativement profond, avec une bonne congruence articulaire.

**Figure 3:**
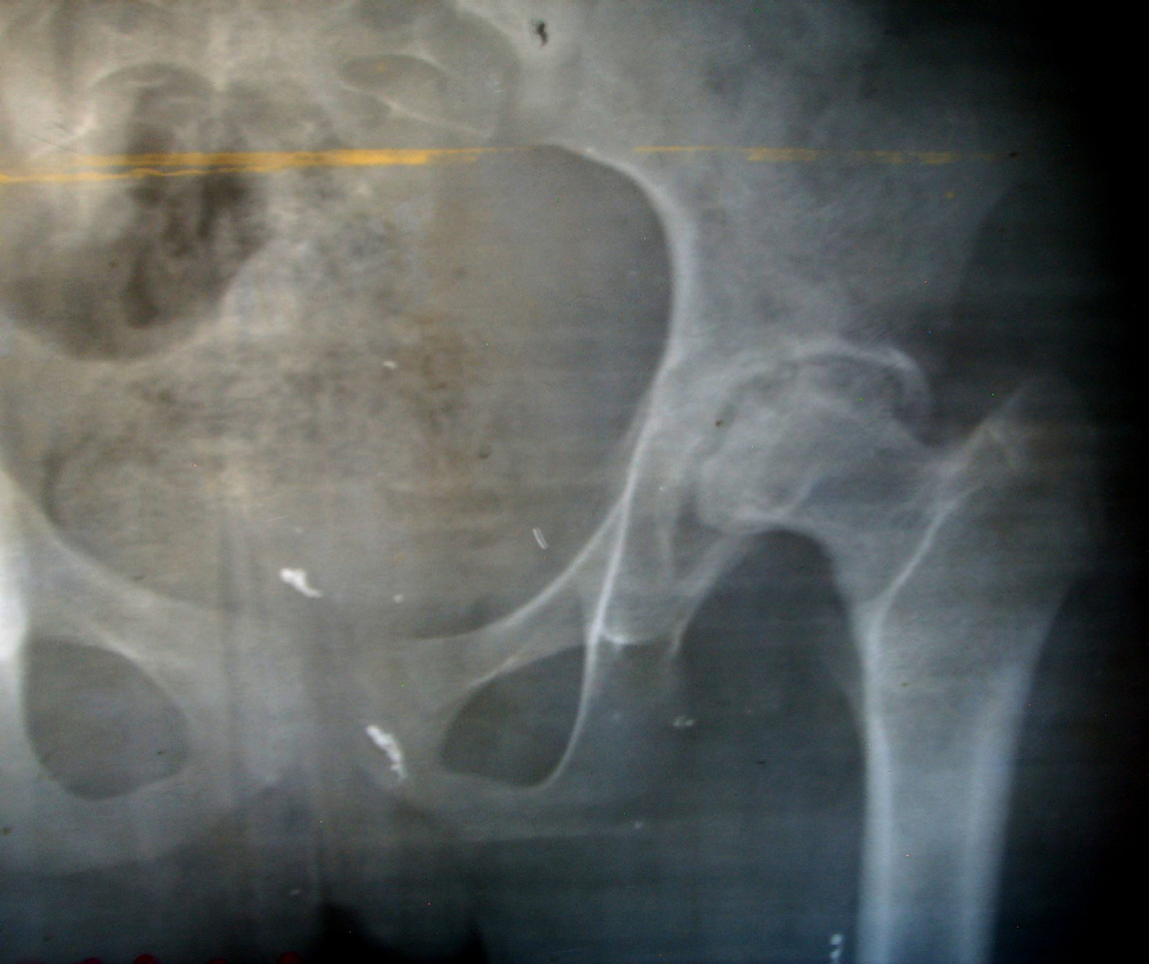
FP (cas n° 2). Séquelle d'injection intrafessière à l’âge de 7 ans. L'enfant est vu à l’âge de 14 ans. Noter la destruction de la tête fémorale, l’évasement du cotyle et la rupture du cintre cervico-obturateur. La tête fémorale reste centrée et bien couverte. FP (case n°2). Sequelae of intragluteal injection performed at the age of 7. The child is seen when he was 14. Notice the destruction of the femoral head, the widening of the acetabulum. The femoral head is still well centered and covered.

**Figure 4:**
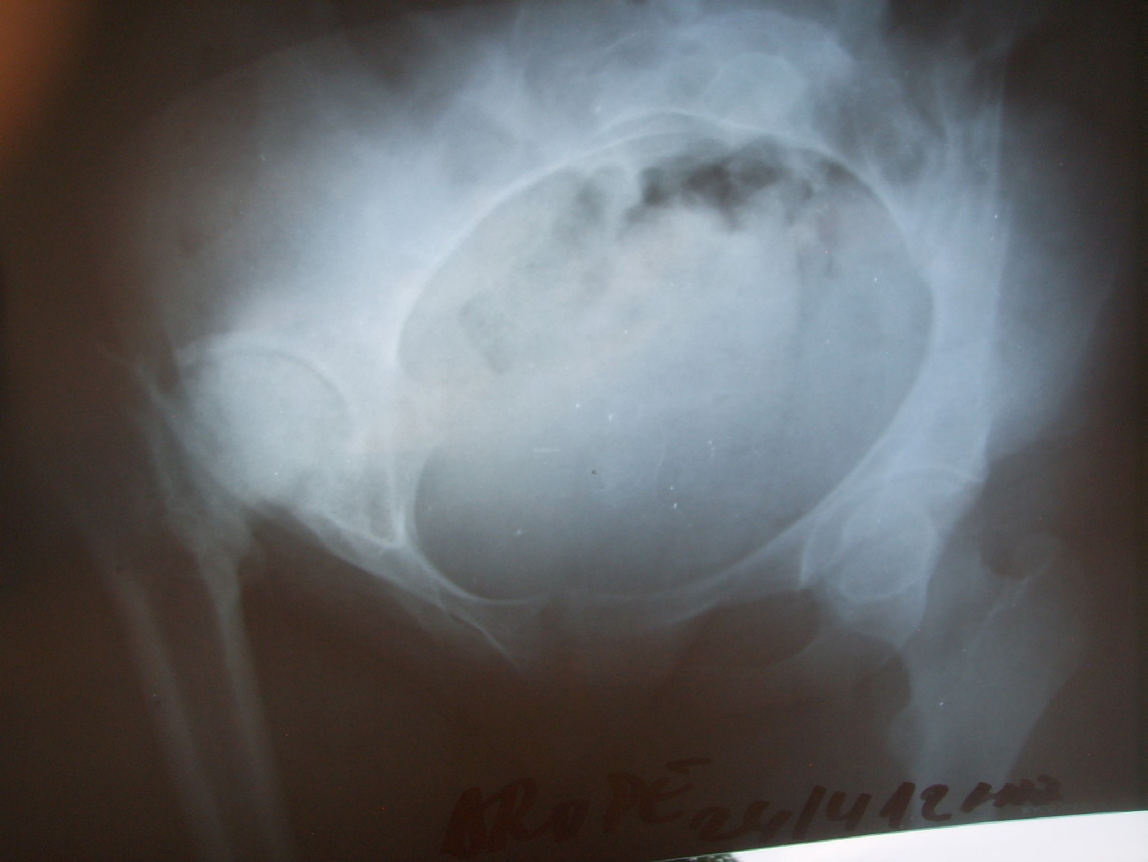
NJ (cas n° 3). Séquelles d'injection intrafessière à l’âge de 12 ans. La patiente est âgée de 18 ans. La hanche droite est raide en adduction, avec une amplitude de flexion de 60°. Le raccourcissement est de 10 cm. Noter la sphéricité de la tête fémorale et son encastrement dans le cotyle. NJ (case n° 3). Sequelae after intragluteal injection performed at the age of 12. The patient is 18 years old. The right hip is stiff in adduction, with a flexion motion of 60°. The shortening is 10 cm. Notice the spherical aspect of the femoral head well-fitted in the acetabulum.

Du fait des conditions matérielles disponibles, les examens paracliniques se sont limités à un bilan radiographique standard. La nécrose intéresse à la fois la tête fémorale et le cotyle et elle est constamment associée à un pincement de l'interligne articulaire. Au niveau du fémur, la nécrose atteint la quasi-totalité de l’épiphyse, mais le moignon épiphyso-métaphysaire garde une forme grossièrement sphérique (Fig. 3). Au niveau du cotyle, on note une ostéolyse diffuse qui intéresse surtout le toit du cotyle; cette ostéolyse est responsable d'une ascension de la tête du fémur qui aggrave le raccourcissement. Mais le cotyle n'est pas dysplasique et la tête fémorale reste centrée. La congruence articulaire est bonne, paraissant liée à la sphéricité du moignon épiphyso-métaphysaire et à l'ossification du bord externe du cotyle, donnant un aspect d'encastrement de l'extrémité supérieure du fémur dans le néocotyle (Fig. 4). La hauteur de l'interligne articulaire est diminuée, l'interligne articulaire est irrégulier, mais il reste présent, suggérant que la toxicité de la quinine atteint surtout le tissu osseux, et dans une proportion moindre le tissu cartilagineux, plus résistant.

Dans un cas (observation n° 4 L. I.), une biopsie synoviale de la hanche a montré « une fibrose organisée, non inflammatoire, renfermant de rares vaisseaux à paroi relativement épaisse ». Au plan thérapeutique, en l'absence de possibilités de reconstruction articulaire, une mise en décharge au moins partielle par l'utilisation de cannes ou béquilles a été conseillée.

## Discussion

Cette étude comporte des limites. L'absence de suivi des patients dans le temps rend impossible une étude longitudinale. La quasi-totalité des patients a été vue quelques années après l'injection responsable, sans document permettant de décrire les premiers stades de la nécrose post-injection. Enfin les conditions matérielles n'ont permis de réaliser que des clichés radiographiques standards.

Au plan épidémiologique, cette étude n'apporte pas de données qui permettraient de connaître l'incidence de la nécrose de la hanche après injection intrafessière de sels de quinine. Il semble cependant que cette complication, qui dans cette série représente 4,4 % des complications observées après injection intrafessière, soit relativement fréquente mais souvent méconnue, probablement confondue avec d'autres étiologies de coxopathie chez l'enfant en milieu africain, notamment la nécrose fémorale sur drépanocytose ou surtout l'arthrite septique de la hanche. Dans son étude épidémiologique portant sur 180 cas de coxopathies observées chez l'enfant sur une période de 7 ans au Bénin, Agossou-Voyèmè [2] ne cite pas la nécrose post injection comme étiologie possible.

La toxicité locale de la quinine est connue depuis longtemps [7, 14]. L'injection intramusculaire de quinine provoque une nécrose musculaire qui favorise l'abcédation en cas de contamination bactérienne [8], surtout en cas d'injections multiples [1]. Une gangrène des membres inférieurs après plusieurs injections intramusculaires de Quinimax^®^ a même été rapportée par Carsalade [5]. L'injection dans la face antérieure de la cuisse peut provoquer une fibrose quadricipitale entrainant une rétraction du muscle bloquant le genou en extension ou même en recurvatum [15]. Une injection à proximité du nerf sciatique provoque une paralysie du nerf, par traumatisme direct, ou liée à la toxicité locale de la quinine [3, 4, 13].

Par contre, on ne retrouve pratiquement pas d’études rapportant une toxicité de la quinine au niveau de l'articulation de la hanche. Kpanodou [12] rapporte un cas de paraostéoarthropathie développée au niveau de la hanche après une lésion du nerf sciatique par injection intramusculaire mais il ne s'agit pas d'une atteinte de l'articulation elle-même. Harouna [10] rapporte un cas d'ostéoarthrite septique apparue après une injection intrafessière, avec atteinte prédominante de l'os iliaque, suggérant que l'infection résultait plus d'une contamination de l'os que de l'articulation elle-même. Cette observation de Harouna est la seule complication ostéo-articulaire post-injection que l'on retrouve dans la littérature. Aucun des six cas de la série ne présentait de paralysie sciatique associée, ce qui est un argument en faveur d'une injection intra-articulaire, le produit ayant été injecté dans l'articulation et n'ayant pas agressé le nerf sciatique.

Au plan clinique, les données de l'anamnèse sont souvent peu fiables. La notion d'une injection intrafessière dans les semaines précédant le début des troubles est toujours retrouvée, soit rapportée spontanément, soit retrouvée à l'interrogatoire, bien que spontanément les familles incriminent souvent un traumatisme comme étant à l'origine des troubles.

Les données de l'examen sont très comparables d'un patient à l'autre: le raccourcissement apparent est toujours accentué, compris entre 6 cm et 10 cm, partiellement compensé par un équinisme à la marche. Le raccourcissement est dû à un raccourcissement vrai lié à la stérilisation du cartilage de croissance métaphyso-épiphysaire et à l’érosion du cotyle: il est aggravé par l'obliquité pelvienne due à l'attitude vicieuse de la hanche en adduction. La hanche est douloureuse, l'abduction est impossible, l'articulation est enraidie en adduction et flexion; dans quelques cas on a noté quelques degrés de mobilité en flexion, mobilité dont l'amplitude ne dépasse jamais 30°.

Dans un contexte africain, ce tableau de coxopathie chez l'enfant peut faire discuter une nécrose ischémique de la tête du fémur, complication fréquente de la drépanocytose [11]. Cependant le tableau clinique de la drépanocytose est plus discret et la nécrose est parfois même asymptomatique [16]. Par ailleurs, l'aspect radiographique de la nécrose drépanocytaire est plus proche de celui de l'ostéochondrite primitive de la hanche. La hauteur de l'interligne articulaire reste normale et l'atteinte du cartilage de croissance est rare. Il n'existe pas d'atteinte du cotyle, sauf tardivement, en rapport avec des remaniements dégénératifs arthrosiques.

L'aspect radiographique et le contexte d'une injection trop profonde peuvent surtout faire évoquer une séquelle d'ostéo-arthrite septique de la hanche, favorisée par la nécrose tissulaire due à la toxicité de la quinine. Certains caractères peuvent faire évoquer ce diagnostic: notion d’état fébrile, d'inflammation locale ou de suppuration, cicatrices de trajets fistuleux. Sur la radiographie, la nécrose épiphysaire est variable, parfois totale. Elle s'accompagne souvent d'une subluxation ou d'une luxation de la hanche, notée dans 44 % des cas par Cottalorda [6] et dans 50 % des cas par Forlin et Milani [9], complication qui semble absente en cas de nécrose post-injection. L'atteinte cotyloïdienne est rare et revêt souvent l'aspect d'une dysplasie.

Les possibilités thérapeutiques sont très limitées chez l'enfant. Au stade évolutif de la nécrose, on ne peut que conseiller une mise en décharge au moins partielle. Une importante adduction sur une hanche totalement enraidie peut faire discuter une ostéotomie d'abduction qui rééquilibre le bassin et diminue le raccourcissement. Au terme de la croissance, une arthroplastie de hanche est à discuter selon les possibilités locales.

## Conclusion

Bien qu'il n'existe pas d'arguments irréfutables permettant d'affirmer le diagnostic de nécrose de la hanche après injection intrafessière de quinine, les tableaux cliniques et radiographiques observés paraissent suffisamment univoques et caractéristiques pour individualiser cette complication et pour attirer l'attention sur sa gravité. Même si elle est peu fréquente, il s'agit d'une complication grave, purement iatrogène, qui justifie pleinement les recommandations de l'OMS de ne plus utiliser cette molécule en intramusculaire pour le traitement du paludisme grave de l'enfant et de la remplacer par l'artésunate qui peut être administré par voie rectale, par voie intramusculaire ou intraveineuse. Dans les cas où seule la quinine est disponible pour traiter une forme sévère de paludisme, la voie intraveineuse est à privilégier.

## Conflits D'intérêts

Les auteurs ne déclarent aucun conflit d'intérêt et aucun financement.
